# SPECT and PET Serve as Molecular Imaging Techniques and *in Vivo* Biomarkers for Brain Metastases

**DOI:** 10.3390/ijms15069878

**Published:** 2014-06-03

**Authors:** Barbara Palumbo, Tommaso Buresta, Susanna Nuvoli, Angela Spanu, Orazio Schillaci, Mario Luca Fravolini, Isabella Palumbo

**Affiliations:** 1 Section of Nuclear Medicine and Health Physics, Department of Surgical and Biomedical Sciences, University of Perugia, Perugia 06100, Italy; E-Mail: tommaso.buresta@libero.it; 2 Section of Nuclear Medicine, Department of Clinical and Experimental Medicine, University of Sassari, Sassari 07100, Italy; E-Mails: susannanuvoli@gmail.com (S.N.); angela.spanu@email.it (A.S.); 3 Department of Biomedicine and Prevention, University of Rome Tor Vergata, Roma 00133, Italy; E-Mail: orazio.schillaci@uniroma2.it; 4 IRCCS Neuromed Pozzilli, Pozzilli (Isernia) 86077, Italy; 5 Department of Engineering, University of Perugia, Perugia 06100, Italy; E-Mail: mario.fravolini@unipg.it; 6 Section of Radiotherapy Department of Surgical and Biomedical Sciences, University of Perugia, Perugia 06100, Italy; E-Mail: isabella.palumbo@unipg.it

**Keywords:** brain metastases, molecular imaging, PET, SPECT, MRI, fusion imaging

## Abstract

Nuclear medicine techniques (single photon emission computerized tomography, SPECT, and positron emission tomography, PET) represent molecular imaging tools, able to provide *in vivo* biomarkers of different diseases. To investigate brain tumours and metastases many different radiopharmaceuticals imaged by SPECT and PET can be used. In this review the main and most promising radiopharmaceuticals available to detect brain metastases are reported. Furthermore the diagnostic contribution of the combination of SPECT and PET data with radiological findings (magnetic resonance imaging, MRI) is discussed.

## 1. Molecular Imaging in Brain Metastases

Molecular medicine represents a strategy that allows to explore the molecular causes of diseases with the aim of contributing to diagnosis and treatment. To control disease evolution before it becomes clearly evident with conventional morphological imaging techniques or laboratory parameters, it is necessary to analyse functional molecular alterations *in vivo* by means of non-invasive, specific molecular imaging modalities providing morpho-functional and biochemical *in vivo* information [1].

Nuclear medicine offers such techniques of molecular imaging by studying the body distribution of radiopharmaceuticals (gamma and positron-emitters) administered to the patient and visualized by SPECT (single photon emission computerized tomography) or PET (positron emission tomography) scanners.

To image brain metastases many radiopharmaceuticals, either gamma or positron emitters, are available. Although contrast-enhanced magnetic resonance imaging (MRI) represents the option of choice to investigate primary and secondary cerebral tumours for the defined anatomical information and spatial resolution [2], nuclear medicine techniques are useful in brain tumours and metastastatic disease to investigate recurrence comparing with effects of treatment (such as radionecrosis following radiotherapy) and to monitor therapy response [2,3,4]. A further and recent indication is represented by the use of nuclear medicine techniques to contribute to the radiotherapy treatment planning.

The possibility to obtain fused images of nuclear medicine and radiology modalities with dedicated softwares or hybrid systems improves diagnostic accuracy. Hybrid systems [SPECT/computerized tomography (CT), PET/CT and PET/MRI] are tomographs combining nuclear medicine (SPECT and PET) and radiology devices (CT, MRI) mounted on the same gantry to obtain co-registered and fused images providing both functional and anatomical information.

In this review the main and most recent radiopharmaceuticals and imaging techniques available in nuclear medicine to detect brain metastases will be evaluated. Positron-emitting radiopharmaceuticals visualized by PET scan will be presented for first, as they represent the most valid and promising biomarkers to investigate brain tumours, while gamma-emitting radiocompounds, visualized by SPECT scan, will be discussed for the particular role played in the past, before the wide diffusion of PET tomographs. Finally findings obtained by multi-modality imaging combining nuclear medicine and radiological images will be reported.

## 2. Positron-Emitting Radiopharmaceuticals

Positron-emitting radiopharmaceuticals represent the principal agents for molecular imaging [1], because they *in vivo* label biochemical processes and metabolic pathways (*i.e*., glycolysis and protein/DNA synthesis), comparing with gamma-emitting radiopharmaceuticals able to indirectly enter in a specific metabolic function (*i.e*., ^201^Thallium, a cardiac perfusion radiocompound, binds the Na^+^/K^+^ ion exchange pump and it is transported into the myocardiocytes). However positron-emitting radiocompounds have some disadvantages, because their production depends on the presence of an on-site cyclotron and the final process is expensive [3,5,6]. Only the radiolabelled compounds with long half-life do not require on-site cyclotron because they can be transported in a different site from the place where they were synthesized and therefore they can be commercially available. Fluorinated compounds are useful and diffuse for the longer half-life of ^18^Fluorine (^18^F, 110 min) with respect to those labelled by other radioisotopes such as ^11^Carbon (^11^C, 20 min) or ^15^Oxygen (^15^O, about 2 min) or ^13^Nitrogen (^13^N, about 10 min) [5]. Furthermore the spatial resolution of PET scan is up to 5 mm, while that of SPECT is about 10 mm [3].

### 2.1. Glucose Radiolabelled Analogue: 2-Deoxy-[^18^F]fluoro-d-glucose (^18^FDG)

Among the group of positron-emitting radiolabelled compounds 2-deoxy-[^18^F]fluoro-d-glucose (^18^FDG), a using glycolytic pathways, is the most widely used because it is available in all the PET Centres. Other more tumour-selective but less diffuse radiopharmaceuticals will be also reported in this review.

**Figure 1:**
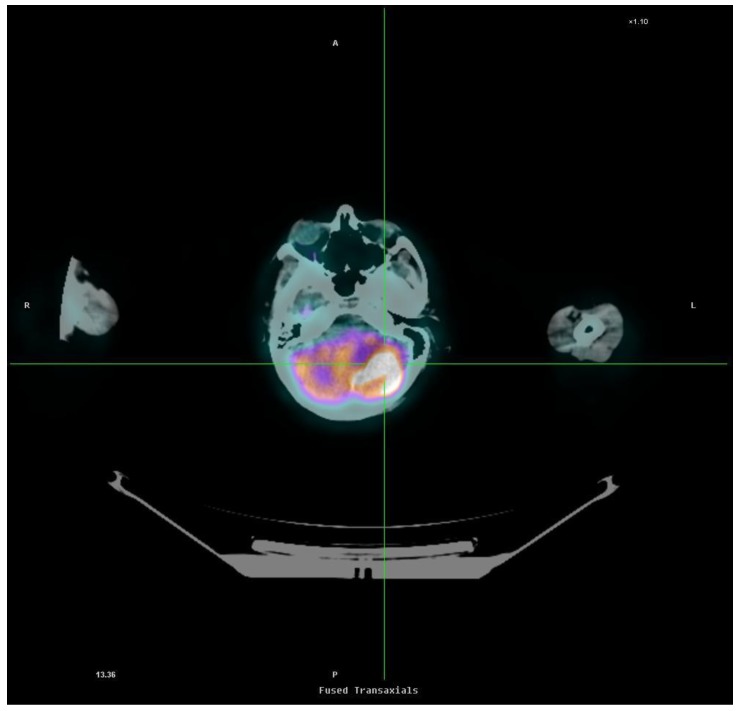
2-Deoxy-[^18^F]fluoro-d-glucose(^18^FDG), positron emission tomography(PET)/computerized tomography (CT) image (transaxial section) of a patient with a metastasis in the left cerebellum deriving from non-small-cell-lung cancer (NSCLC). The metastasis appears as an area of hyperfixation of ^18^FDG in the context of normal cerebellar parenchyma with homogeneous and symmetrical radiopharmaceutical distribution.

The uptake mechanism of ^18^FDG in tumour cells depends on the increased number of functional glucose transporters and glycolytic enzymes [5]. ^18^FDG is physiologically and homogeneously distributed in both cerebral and cerebellar hemispheres (glucose avid) and brain tumours are visualized as lesions with higher or lower radiopharmaceutical uptake comparing with normal brain parenchyma [3]. An example of a cerebellar metastasis deriving from lung tumour imaged by ^18^FDG PET/CT is reported in Figure 1.

Although ^18^FDG PET was carried out to investigate the presence of brain tumours and metastases in many studies, the need to detect lesions with higher or lower radiopharmaceutical uptake with respect to normal brain parenchyma (with physiologic glucose uptake) and the resolution power of PET scan (5 mm), limit the use of this radiocompound [3,4]. A retrospective analysis of the main studies concerning the role of ^18^FDG PET in brain metastases is reported as follows.

Lee *et al*. [7] evaluated 48 patients with lung cancer having brain metastases investigated by MRI. All patients underwent ^18^FDG whole body and brain PET to evaluate primary lung tumour and brain metastases. The authors showed that 33% of the brain lesions detected by MRI were not clearly detected by ^18^FDG PET, even though primary lung tumours showed intense ^18^FDG uptake. The authors reported that when the primary lesion of lung cancer had hyperfixation of ^18^FDG, radiopharmaceutical uptake in metastatic brain lesions was variable. One-third of brain metastases from lung cancer had hypometabolism (lower ^18^FDG accumulation). Non-small-cell lung cancer (NSCLC) patients had more frequently hypermetabolic (higher ^18^FDG uptake) brain metastases than small-cell-lung cancer (SCLC) subjects (80% and 26.7% respectively, *p* < 0.01).

In an interesting study Krüger *et al*. [8] prospectively recruited 104 neurological asymptomatic patients with primary diagnosis of lung cancer undergoing ^18^FDG cerebral PET/CT and brain MRI. MRI was able to detect overall 100 brain metastases, while PET/CT only 17 (*p* < 0.01). PET/CT showed a sensitivity of 27.3%, a specificity of 97.6%, a positive predictive value (PPV) of 75% and a negative predictive value (NPV) of 83.3% in diagnosing brain metastases. The authors concluded that PET/CT comparing with the gold standard MRI missed many lesions in many patients and was less accurate for detecting multiple and smaller brain metastases.

A previous paper of Wang *et al*. [9] reported a ten years’ experience concerning ^18^FDG-PET on brain tumours treated by radiotherapy, evaluating the contribution of PET in the differential diagnosis between recurrence and radiation therapy effects. The authors evaluated 156 PET scans of 117 patients with primitive and secondary brain tumours with positive but not diagnostic MR and/or CT. PET data were correlated to histopathologic findings and clinical follow up. The positive predictive value of PET resulted 96% in all subjects and 100% in brain metastases of lung carcinoma, while the negative predictive value basing on histopathologic examination was 55.6%. Furthermore the authors observed that survival was significantly increased in patients with negative PET.

In a recent paper Horky *et al*. [10] investigated the ability of dual phase ^18^FDG-PET/CT to differentiate tumour and radionecrosis in patients treated by stereotactic radiosurgery for brain metastases. Dual-phase ^18^FDG-PET/CT was carried out in 32 consecutive patients with treated brain metastases, lesion size greater than 0.5 cm and suspected recurrence on MR. Maximum and mean standaridized uptake values (SUV_max_ and SUV_mean_ respectively) of the lesion (L) and gray matter (GM) at the level of the thalamus were measured on early and delayed images. SUV ratio provides a measurement of radiopharmaceutical uptake in a certain lesion and is expressed by the following equation: Activity at a pixel (kBq/cm^3^)/injection dose (MBq)/weight (kg) [11]. Horky *et al*. [10] showed that a change higher than 0.19 of L/GM ratios as a function of time had a sensitivity of 95%, a specificity of 100% and an accuracy of 96.4% (*p* = 0.0001; AUC = 0.97) in diagnosing tumour with respect to radiation necrosis. The ratio of the change of the lesion to white matter ratios over time was the second best indicator of outcome comparing with the other indices [receiver operating characteristic (ROC) cut-off = 0.25, sensitivity 89.5% and specificity 90.9%, and accuracy 89.2%; *p* = 0.0001; AUC = 0.95], while early or late SUVs of the lesion alone did not distinguish between tumour and necrosis. Furthermore differentiation between necrosis and metastatic brain disease was improved by using the change of lesion to gray matter SUV_max_ ratios as a function of time independently of histological tumour type. The limit is the long time interval between PET scans (range, 2–5.7 h) as suggested also by other authors [4].

The limits of ^18^FDG can be in part solved by comparing PET functional data with morphological MRI findings and obtaining fused images [12,13]. Results obtained in such studies will be discussed in a following section of this review concerning mutli-modality imaging.

### 2.2. Tumour-Selective Radiopharmaceuticals: ^11^C-Methyl-methionine (MET) and Radiolabelled Choline Compounds

Other radiopharmaceuticals more tumour-specific than ^18^FDG are available. Among these, radiolabelled choline compounds and ^11^C-methyl-methionine (MET) have been used in primary and metastatic brain tumours [3].

Choline is a cellular membrane phospholipid precursor increased in brain tumours and it is radiolabelled by ^11^C and ^18^F. ^11^C-Cho was the first radiocompound synthetized as metabolic substrate for PET imaging, but the more recent fluorinated choline analogues have the advantage of the longer half-life of ^18^F (110 min) with respect to that of ^11^C (20 min) [5]. This makes ^18^F-Cho easy to be produced far from the hospital and commercially available. Radiolabelled choline compounds are mainly used to diagnose prostate cancer [14,15]; furthermore they have been successfully employed to investigate primary brain tumours [16,17,18], but relatively scarce are the studies evaluating radiolabelled choline compounds in detecting brain metastases. The available studies will be presented as follows.

Mertens *et al*. [19] evaluated the optimal timing for imaging brain tumours and other brain lesions with ^18^F-Cho PET. The authors performed dynamic PET imaging (consisting of scans carried out 5–10 min after tracer administration for an acquisition time of 28 min) in 24 patients with space-occupying lesions in the brain. ^18^F-Cho uptake ratios were calculated by measuring indices of lesion tracer uptake to normal tissue (LNRs) after coregistering PET and MRI images to obtain a correct alignment. Time-activity curves (TACs) were generated basing on LNRs and they expressed radiopharmaceutical uptake over time. Findings obtained in gliomas of different grading showed that, after a rapid tracer uptake phase, the mean increase in LNRs was 1.07 ± 0.93 for glioblastomas, −0.52 ± 1.56 for anaplastic astrocytomas, 0.04 ± 0.13 for grade 2 oligoastrocytomas and 0.37 in a case of a pilocytic astrocytoma. ^18^F-Cho uptake was also rapid in a metastatic brain lesion from NSCLC and in 2 mass lesions diagnosed as radiotherapy effects, showing a similar average increase in LNR, resulting 0.46 for the brain metastasis and 0.41 ± 0.69 for the radiation-induced mass lesions, while a tumefactive demyelinating lesion presented 1.07. Therefore the authors concluded that ^18^F-Cho dynamic PET scan providing TACs and LNRs measurements represent the preferred PET technique for the detection of brain tumours and other brain lesions.

Another interesting paper of Rottenburger *et al*. [20] compared MET and ^11^C-Cho-PET results in 8 patients with brain metastases (confirmed in 7 of them). MET, a methionine analogue labelled by ^11^C, belongs to the group of the radiolabelled amino acids. These radiopharmaceuticals are specific to image tumours because their uptake is mediated by increased amino acid transport across cell membrane and incorporation into proteins, thus reflecting protein synthesis rate that is increased in neoplastic disease [3,5].

Rottenburger *et al*. [20] in their study reported that mean SUV values of the lesions were in median 1.8 for MET and 1.1 for ^11^C-Cho, median lesion-to-normal brain tissue ratios (LNRs) were 1.5 for MET and 6.6 for ^11^C-Cho. ^11^C-Cho LNRs were significantly higher than those of MET (*p* = 0.007). Therefore the authors concluded that ^11^C-Cho seemed to be promising to image brain metastases comparing with MET, for the significantly higher LNRs in tumour tissue with respect to those of MET and for the evidence of a decreased specificity.

In animal models, it has been demonstrated that the upregulation of the amino acid transporter in the supporting vasculature of brain tumour tissue determines increased facilitation of amino acid transport into the tumour cells [21]. The factors involved in this active transport are the flux of the amino acid to the tissue, the intrinsic activity of the amino acid transporter and the rate of intracellular amino acid metabolism [22]. Amino acid transport is generally considered as the rate-limiting step, also for the few amino acid tracers that are incorporated into the proteins [23].

^11^C-Cho and amino acid labelled analogues, such as MET, are able to selectively evidence the lesion as a hyperactive (“hot”) area in the context of a normal brain parenchyma with no radiocompound uptake. MET is also useful in investigating the differential diagnosis between recurrent tumour and radionecrosis both in primary and secondary brain tumours and tumour-to-lesion uptake ratios can contribute to the diagnostic process [24,25]. However it has to be reminded that small metastases can be identified only by MRI and that an unspecific uptake in inflammatory process has been described in MET-PET scan in comparison with imaging obtained with other radiopharmaceuticals [24,26]. False-positive results due to unspecific radiopharmaceutical uptake were observed in patients studied by MET-PET in case of necrosis, leucoencephalitis, brain abscesses, haematoma, ischaemia and demyelination [24]. This potential lack of selectivity for tumours might lead to prefer other more specific radiopharmaceuticals, but MET is widely considered as a valuable tracer to image brain tumours and metastases for the meaningful data described by international literature.

Terakawa *et al*. evaluated by MET-PET 51 patients with brain metastases and 26 with glioma after radiation treatment, reporting lower tumour-to-lesion uptake ratios in patients with recurrent metastases than glioma [27]. Also Grosu *et al*. showed higher mean T/N ratio in patients with treated glioma (2.6 ± 0.9), as compared with those with treated metastases (1.8 ± 0.2) [28].

Yamane *et al*., in 30 patients with treated metastatic brain tumours, observed a high diagnostic accuracy to differentiate recurrent metastases and radiation therapy effects [25].

Furthermore the use of MET-PET is very promising as a diagnostic tool for radioterapy treatment planning in order to obtain a biological target volume (BTV), representing the biologically aggressive part of the tumour that requires to be mostly irradiated.

Miwa *et al*. [29] evaluated 42 metastatic brain tumours at baseline, 3 and 6 months after stereotactic intensity-modulated radiation therapy (SRT-IMRT). In this study MET-PET images were imported in the planning software for the SRT-IMRT dosimetry as additional information and the final target volume was defined and drawn on the stereotactic MR image, taking into account MET-PET and MRI findings. MET-PET was useful to detect the characteristic changes following SRT-IMRT. Mean LNRs MET uptake ratios were 1.95 ± 0.83 at baseline, 1.18 ± 021 three months after treatment (*p* < 0.0001 *vs*. baseline) and 1.12 ± 0.25 six months after (*p* < 0.0001 *vs*. baseline), thus suggesting that MET uptake decrease could be secondary to metabolic changes. The authors concluded that MET-PET was useful in providing additional information for diagnosis and follow up after radiotherapy.

Matsuo *et al*. [30] evaluated 19 patients for a total of 95 brain metastatic lesions. MET-PET and MRI were carried out to contribute to the gross tumour volume (GTV) for the radiotherapy treatment planning evidencing that MET-PET has the potential for the precise delineation of target volumes particularly in lesions with a volume higher than 0.5 mL.

Tang *et al*. reported the development of a threedimensional gaussian model that was helpful for determination of bulk tumour limits in 20 patients with metastases [31].

### 2.3. ^18^F-Labeled Aromatic Amino Acid Analogues: O-(2-^18^F-Fluoroethyl)-l-tyrosine (FET) and 3,4-Dihydroxy-6-^18^F-fluoro-l-phenylalanine (^18^F-DOPA)

Other radiopharmaceuticals used in oncology are represented by ^18^F-labeled aromatic amino acid analogues, such as *O*-(2-^18^F-fluoroethyl)-l-tyrosine (FET) and 3,4-dihydroxy-6-^18^F-fluoro-l-phenylalanine (^18^F-DOPA) [23,28]. These radiocompounds have a brain tumour uptake similar to that of ^11^C-methionine [23] with the advantage of the longer half-life with respect to ^11^C-labelled compounds.

In an interesting study of Stober *et al*. [26] human tumour and inflammatory cells were incubated with 370 kBq of FET or 3.7 kBq of MET for 15 min to investigate the different uptake of the radiopharmaceuticals. Kinetic studies were carried out at variable concentrations of FET and MET and competitive inhibition studies were performed with competitive inhibitors of the main neutral amino acid transport systems (2-amino-2-norbornane carboxylic acid for the L system, α-(methylamino) isobutyric acid for the A system and l-serine for the ASC system). MET had a significantly higher uptake in all cells examined with respect to FET; furthermore inflammatory cells incorporated more MET than tumour cells, while FET uptake was significantly higher in tumour cells than in the inflammatory ones. In the tumour cells FET and MET uptake seemed to be mediated almost exclusively by the mentioned specific transport systems, because radiocompound uptake was decreased to below 10% when applying all three inhibitors. In inflammatory cells only 50% of the radiopharmaceutical uptake was inhibited by the combined transport inhibitors. Therefore the high degree of “unspecific uptake” in inflammatory cells might be due to the presence of unknown transport systems resistant to the common inhibitors or uptake via free diffusion or pinocytosis. In conclusion, FET accumulated to a significantly greater extent in tumour than in inflammatory cells comparing with MET, thus supporting the use of FET in oncology. The differences between tumour and inflammatory cells concerning FET and MET uptake suggested that these radiopharmaceuticals represent substrates of different subtypes of the L system.

Grosu *et al*. in an interesting paper [28] carried out on the same day both FET-PET and MET-PET in 42 patients, 29 affected by pretreated gliomas and 13 by brain metastases, to obtain an interindividual comparison of the diagnostic performance of the two radiocompounds. Quantification of uptake of FET and MET was obtained by SUVs. Both radiopharmaceuticals were able to differentiate tumour and treatment-related changes with a sensitivity of 91% and a specificity of 100%. The authors concluded that FET-PET and MET-PET provided comparable diagnostic information on gliomas and brain metastases, particularly in the differentiation of residual/recurrent tumour from treatment-related changes/pseudoprogression. Furthermore the authors performed by MET-PET and FET-PET mean GTV in 17 glioma patients; Results were not significantly different using both tracers, thus appearing as promising radiocompounds to contribute to the treatment planning.

Galldicks *et al*. in a subsequent paper [2] investigated the ability of FET PET to differentiate local recurrent brain metastasis and radiation necrosis in 31 patients with 40 secondary brain lesions. It has been shown that although contrast-enhanced MRI is the method of choice to evaluate primary and secondary brain tumours, it is not always capable to provide conclusive data in case of non-specific inflammatory reaction caused by irradiation, tumour necrosis or postoperative enhancement along the resection margins [32]. Galldicks *et al*. [2] performed dynamic FET PET consisting of image acquisition up to 50 min after radiopharmaceutical intravenous injection. Maximum and mean tumor-to-brain ratios of FET uptake (TBR_max_ and TBR_mean_, respectively) obtained 20–40 min after tracer injection were evaluated, time–activity curves were generated and the time to peak (TTP) was measured. TBR_max_ and TBR_mean_ resulted significantly higher (*p* < 0.001) in patients with recurrent metastases (No. 19) comparing with patients with radiation necrosis (No. 21). The diagnostic accuracy of FET PET to identify recurrent brain metastases was 78% using TBR_max_ and 83% using TBR_mean_. Accuracy was also increased with different pattern of time-activity curves. The authors concluded that the combination of the TBR_mean_ of FET uptake and the pattern of the time–activity curve was useful in the differential diagnosis between local brain metastasis recurrence and radionecrosis, thus affirming that FET PET can provide a valid contribution to the management of patients with brain metastases.

A further useful amino acid radiopharmaceutical is ^18^F-DOPA, that has been successfully used to image brain tumours due to high tumour uptake with respect to normal brain parenchyma for the increased neoplastic amino acid transport [33]. Although interesting studies reported the clinical utility of ^18^F-DOPA in primary brain tumours, either in diagnosis or to contribute to the treatment planning, scarce are the data available on metastases [33,34,35]. To our knowledge only 3 studies included in the group of patients few subjects with brain metastases [36,37,38]. Becherer *et al*. [36] in a paper comparing ^18^F-DOPA and MET in a group of 20 patients (18 with brain tumours, 1 with brain metastasis and 1 with non-cerebral tumour) evaluated a single brain metastases deriving from NSCLC showing uptake of both ^18^F-DOPA and MET. Jacob *et al*. [37], in a study comparing ^18^F-DOPA and ^18^FDG PET scan in four patients with newly diagnosed SCLC, observed also a single brain metastatic area accumulating ^18^F-DOPA. Furthermore in the most recent paper Schiepers *et al*. [38] investigated 37 individuals with brain tumours: Among them only two had brain metastatic lesions from somatic cancer and showed intense pathological radiopharmaceutical uptake. As suggested by Calabria *et al*. [33] ^18^F-DOPA can be considered useful in investigating brain metastases for the ability to cross the blood brain barrier, the low distribution in normal gray and white matter and the high tumour to normal tissues ratios; however the majority of the studies were focused on evaluating primary brain tumours, as they represent an important diagnostic challenge in the differential diagnosis between recurrence and radionecrosis. To our knowledge no technical problems limit the use of ^18^F-DOPA PET in brain metastases, but this tool was more widely employed to evaluate primary tumours in available international literature.

A further interesting radiopharmaceutical is 3'-deoxy-3'-^18^F-fluorothymidine (^18^FLT) able to *in vivo* image proliferation because it is a thymidine analogue that is intracellulary phosphorylated by the thymidine kinase 1 (TK1) in its nucleotide monophosphate that it is not further metabolized and therefore it is accumulated in the cells. TK1 is a cytosolic enzyme expressed with the onset of S phase during DNA synthesis. TK1 has a higher activity in proliferating tumour cells with respect to normal tissue and therefore ^18^FLT uptake represents an in vivo biomarker of proliferation acitivity of tissues [39]. ^18^FLT has been used as a radiopharmaceutical to image brain tumours and other systemic tumours, but also in the case of this tracer, few data are available in literature concerning the study of brain metastases. Schiepers *et al*. [40] investigated 7 patients with high-grade tumors and 2 with metastases by means of dynamic ^18^FLT PET images acquired after tracer administration for 75 min to obtain a three-compartment model with blood volume, metabolite, and partial volume corrections in order to evaluate radiopharmaceutical kinetics in malignant brain tumors. The standard three-compartment model resulted appropriate to describe ^18^FLT uptake and corrections for blood volume, metabolites, and partial volume were necessary. Kinetic data were correlated with tumour pathology and clinical follow-up. On the basis of the analysis two groups of lesions were distinguished: Tumour predominant (TumP) and treatment change predominant (TrcP). TrcP group showed a relatively low *k*_3_ (expressing phosphorylation rate), being significantly reduced with respect to that of TumP group (*p* < 0.01). The fraction of transported ^18^FLT that was phosphorylated [expressed by the formula *k*_3_/(*k*_2_ + *k*_3_)] was capable to separate the two groups (*p* < 0.001). The authors concluded that a three-compartment model with blood volume, metabolite and partial volume corrections may adequately describe ^18^FLT kinetics in malignant brain tumours. Furthermore patients could be divided as having tumour-predominant or treatment change-predominant lesions, showing significantly different phosphorylation rates.

## 3. Gamma-Emitting Radiopharmaceuticals

Finally, although positron-emitting radiopharmaceuticals represent the ideal *in vivo* biomarkers to image brain tumours and metastases, for the mentioned ability to *in vivo* label biochemical and metabolic pathways the gamma-emitting radiocompounds selective for neuro-oncology played a relevant role in the past and can be still used. Gamma-emitting radiopharmaceuticals are visualized by SPECT scan and are commercially available, thus not requiring the presence of the on-site cyclotron. However the resolution power of SPECT is lower than that of PET tomograph. Many radiopharmaceuticals have been used to study brain tumours: ^201^Thallium (^201^Tl), ^99^ ^m^Tc-sestamibi (^99^ ^m^Tc-MIBI), ^99^ ^m^Tc-tetrofosmin and ^123^I-iodine-α-methyl tyrosine (^123^IMT) [3,32,41].

### 3.1. Cardiac Radiopharmaceuticals: ^201^Thallium (^201^Tl), ^99^ ^m^Tc-Sestamibi (^99^ ^m^Tc-MIBI), ^99^ ^m^Tc-Tetrofosmin

^201^Tl, ^99^ ^m^Tc-MIBI and ^99^ ^m^Tc-tetrofosmin are commonly used as cardiac radiocompounds to image myocardial perfusion. ^201^Tl is an element belonging to the group IIIA, but its behaviour is chemically similar to potassium. ^201^Tl uptake in brain tumours is a result of a combination of factors, such as alterations in the blood brain barrier, variability in the expression of the Na^+^/K^+^ pump (viable cell having intact uptake mechanism) and blood flow. ^99^ ^m^Tc-MIBI and ^99^ ^m^Tc-tetrofosmin accumulate in cytoplasm and mitochondria due to a passive diffusion across the negative cellular/organelle membrane [3]. In the past cardiac radiopharmaceuticals have been widely used as neuro-oncological radiotracers, being able to be selectively taken-up by brain tumours comparing with normal brain tissue, because they are more avidly incorporated by active proliferating cells [3,5,6,32,41]. More recently the availability of more resolute and tumour-selective positron-emitting radiocompounds lead to a decreased use of gamma-emitting cardiac radiotracers.

### 3.2. Amino Acid Analogue: ^123^I-Iodine-α-methyl tyrosine (^123^IMT)

Among gamma-emitting radiopharmaceuticals ^123^IMT maintains a certain role for its intrinsic properties, although it is not widely available. ^123^IMT shares similar properties with positron-emitting amino acid tracers and is taken up by a stereoselective active transport system across blood brain barrier and brain cell membranes; therefore, although the radiocompound is not clearly incorporated into cellular proteins, its uptake shows a relationship to cell proliferation [3].

Finally our opinion is that positron-emitting radiocompounds represent the option of choice if available with respect to gamma-emitting tracers.

## 4. Multi-Modality Imaging

**Figure 2:**
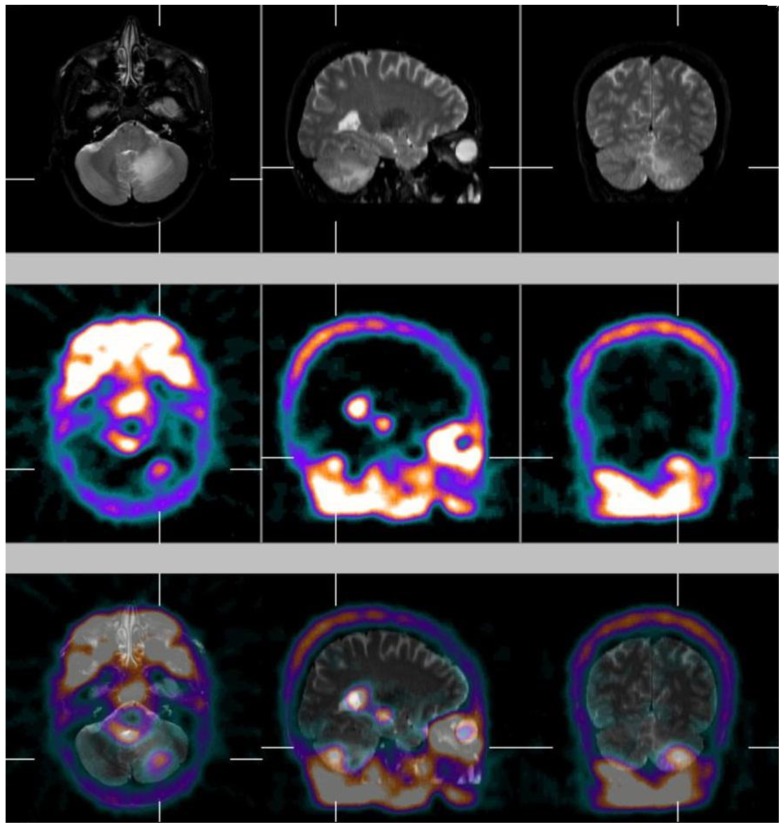
Magnetic resonance imaging (MRI) (**at the top**), ^99^ ^m^Tc-MIBI SPECT (**in the middle**) and fused image of ^99^ ^m^Tc-MIBI single-photon emission computerized tomography (SPECT)and MRI (**at the bottom**) in transaxial, coronal and sagittal sections of a metastasis in the left cerebellum deriving from non-small-cell lung cancer(NSCLC, same patient of Figure 1). The left cerebellar metastasis appears as an area of hyperfixation of ^99^ ^m^Tc-MIBI in the context of normal cerebellar parenchyma without radiopharmaceutical distribution. MRI shows the morphological alteration and the precise anatomical site of the metastasis. Fusion imaging SPECT/MRI allows to anatomically locate the position of the metastasis by MRI, evidenced as an area of selective radiopharmaceutical uptake by ^99^ ^m^Tc-MIBI SPECT images.

At the end the role of the multi-modality imaging has to be mentioned. The association of different modalites such as nuclear medicine and radiology techniques to obtain comparative or fused/hybrid images plays a significant role. Fusion imaging, particularly if it is obtained by means of hybrid systems, that co-register and fuse molecular (PET and SPECT) and morphological (CT, MRI) images, increases the diagnostic accuracy of brain tumours and metastases, in particular because brain is an easy target for image fusion for the rigidity and consistency of bony landmarks, hollow airspaces and soft tissues within skull, thus allowing to obtain perfectly fused brain images of high quality [3]. Figure 2 shows an example of fused image of ^99^ ^m^Tc-MIBI brain SPECT and MRI of a cerebellar metastasis from lung cancer of the same patient showed in Figure 1.

Furthermore MRI may add also morpho-functional information if functional techniques are used, such as magnetic resonance spectroscopy (MRS) diffusion weighted MRI (DWI) with Apparent diffusion coefficient maps (ADC) and perfusion-weighted MRI (PWI) [3,4,12,32]. Data obtained by nuclear medicine techniques (SPECT and PET) and MRI can be considered as complementary and their association may provide more accurate diagnosis of brain tumours either primary or secondary [3,12,32]. In the following part a retrospective analysis of the main studies concerning multi-modality imaging in evaluating primary and secondary brain tumours is reported.

### 4.1. Review of the Main Studies Available in International Literature

Palumbo *et al*. [12] investigated 15 patients with 18 metastatic lesions of tumours of different origin. All the patients underwent ^18^FDG PET, gadolinium-enhanced fast spoiled gradient echo (Gd-FSPGR) and DWI with ADC maps. DWI allows non-invasive grading of tumor cellularity basing on the principle that molecules in living tissues routinely undergo random (Brownian) motion and ADC maps reflect microscopic water diffusibility with respect to factors restricting diffusion within tissue (*i.e*., cell membranes, viscosity). Spherical control regions of interest (ROIs) were selected on Gd-FSPGR images including each tumour lesion and an identical contralateral area of normal brain parenchyma. The ROIs were co-registered by a specific software in the ADC and PET images and Gd-FSPGR represented the target for statistical parametric mapping after realignment of ADC maps and PET images. Ratios were obtained by measuring ^18^FDG uptake and ADC values in tumour and control ROIs. An inverse correlation (*r*^2^ = 0.2746, *p* = 0.0256) was found between ^18^FDG PET and ADC ratios. This could be explained because hypercellular proliferating areas could increase impedance to water diffusion, resulting in low ADC values and high ^18^FDG uptake. The interesting finding of this paper was that the integration of two different modalities (^18^FDG PET and DWI with ADC maps) revealed different aspects of the same biological phenomenon improving diagnosis and clinical management of brain metastatic lesions.

A further interesting example of multi-modality imaging is represented by the paper of Kamson *et al*. [42], that evaluated the accuracy of α-[^11^C]methyl-l-tryptophan (AMT)-positron emission tomography (PET) to differentiate newly diagnosed glioblastomas and brain metastases in 36 adults with suspected brain malignancy. AMT is an amino acid positron-emitting promising radiopharmaceutical tracer able to measure tryptophan metabolism via the immunomodulatory kynurenine pathway [43]. Although this tracer is promising it is scarcely used because it is not widely available. Recent studies demonstrated that AMT accumulates for both transport and metabolism in untreated and recurrent gliomas [43,44]. Kamson *et al*. [42] measured tumour AMT accumulation by SUVs and radiopharmaceutical kinetic analysis was carried out to separate tumoral net tryptophan transport [by AMT volume of distribution (VD)] from unidirectional uptake rates by means of dynamic PET and blood input function. The differential accuracy of these PET variables was investigated and compared with conventional MRI. For glioblastoma/metastasis differentiation, tumoral AMT SUV presented the highest accuracy (74%) and the tumor/cortex VD ratio disclosed the highest positive predictive value (82%); the combined accuracy of MRI (providing size of contrast-enhancing lesion) and AMT-PET was up to 93%. For ring-enhancing lesions, tumour/cortex SUV ratios were higher in glioblastomas comparing with metastatic tumours and were capable to differentiate these two tumour types with great accuracy (higher than 90%). These findings demonstrated that AMT PET can contribute to enhance pretreatment differentiation of glioblastomas and metastatic brain tumors and it may be particularly useful in patients with a newly diagnosed solitary ring-enhancing mass detected by MRI.

Finally Kickengereder *et al*. [4] reported a case-report on the long-term follow-up of a patient treated by stereotactic radiosurgery (SRS) for brain metastastatic disease deriving from lung cancer. Fifty-eight months after SRS, the differential diagnosis between local recurrent brain metastasis and radiation-induced changes by using MRI was unclear. PWI, MRS and MET-PET were performed to contribute to obtain final diagnosis. PWI and MRS data were not conclusive for artifacts and technical limitations, while MET-PET images suggested radiation-induced changes. A subsequent stereotactic biopsy for histological examination evidenced radiation-induced necrosis without tumour. This paper is interesting because in addition to the case-report a detailed review of the literature was also provided. The authors affirmed that beyond structural MRI advanced techniques, such as PWI and MRS, can be successfully used to distinguish recurrent brain metastasis from radiation-induced changes. The authors stated that PWI in particular can be included in the daily practice because the acquisition time is short (less than 10 min) and the sensitivity and specificity are high (varying respectively from 70% to 100% and from 95% to 100% in several studies) [4]. On the other hand also MRS was successfully used to contribute to differentiate brain tumours and radiation induced changes and if associated with nuclear medicine techniques, *i.e*., SPECT and PET, diagnostic accuracy was improved [3,32], thus suggesting it has to be included also in daily clinical practice.

## 5. Conclusions

In conclusion nuclear medicine techniques represent molecular imaging tools able to provide *in vivo* biomarkers of primary and metastatic brain tumours. PET with positron emitting tumour selective radiopharmaceuticals represents the option of choice to obtain the best diagnostic accuracy. ^18^FDG PET can be useful if brain lesions show clearly higher or lower tracer uptake comparing with normal tissues or if fused images with radiological tools providing anatomic location of tumour are available. The association of nuclear medicine and radiological modalities can contribute to improve diagnostic accuracy by combining morphological and functional information.

Finally it has to be remarked that in international literature many papers are available on the role of molecular imaging modalities in detecting primary brain tumours, while data concerning brain metastases are relatively scarce, although the published papers present very promising results. More research works are necessary to support these findings.
